# Annals of Surgical Oncology Landmark Series Disparities in Surgical Oncology: Breast Cancer

**DOI:** 10.1245/s10434-025-18157-0

**Published:** 2025-09-03

**Authors:** Elizabeth M. De Jesus, Leisha C. Elmore, Oluwadamilola M. Fayanju

**Affiliations:** 1 Division of Breast Surgery, Department of Surgery, Perelman School of Medicine, The University of Pennsylvania, Philadelphia, PA USA; 2 Penn Center for Cancer Care Innovation, Abramson Cancer Center, Philadelphia, PA USA; 3 Rena Rowan Abramson Cancer Center, Penn Medicine, Philadelphia, PA USA; 4 Leonard Davis Institute of Health Economics (LDI), The University of Pennsylvania, Philadelphia, PA USA

## Abstract

Breast cancer mortality rates have declined steadily over the past 40 years, as enhanced screening has shifted the balance of new diagnoses to curable, early-stage cancers while advances in systemic therapy have yielded improved and increasingly durable outcomes even for locally advanced and metastatic disease. However, this reduction in mortality has not been equitably distributed among all racial groups, and there continue to be significant racial and ethnic disparities. In this review, we summarize research that has contributed to our understanding of disparities in breast surgical oncology across all racial/ethnic groups, however, given that the most egregious disparities exist among Black women, our review will involve considerable focus on this racial group. We will discuss racial disparities across the breast cancer continuum including access to care, diagnosis, surgical treatment, and survivorship and will conclude with strategies to redress them.

Breast cancer mortality rates have declined steadily over the past 40 years, as enhanced screening has shifted the balance of new diagnoses to curable, early-stage cancers while advances in systemic therapy have yielded improved and increasingly durable outcomes even for locally advanced and metastatic disease. However, this reduction in mortality has not been equitably distributed among all racial groups, and there continue to be significant racial and ethnic disparities.

A health disparity is an observed, potentially avoidable, inter-group difference in health care access, treatment, and/or outcomes related to unjust distribution of societal, economic, and/or environmental sources.^1–6^ Health disparities typically are observed among marginalized groups defined along demographic dimensions including race, ethnicity, religion, sex, age, disability, geographic location, socioeconomic status (SES), or sexual orientation.^5,7^ The etiology of observed disparities often is complex and multifactorial, as evidenced by the fact that lower SES is associated with worse outcomes after breast cancer diagnosis, but racial disparities in survival persist even after adjustment for SES and stratification by stage at diagnosis.^8^

The breast cancer mortality rates among American Indian/Alaskan Native women remain 6 % higher than among white women despite a 10 % lower incidence.^1–3^ The mortality rates are comparable between Asian Americans and white women, but disaggregation of this diverse, heterogeneous group shows more pronounced disparities, particularly for under-represented and disadvantaged groups, including Southeast Asians and Pacific Islanders.^9,10^ A comparison of breast cancer mortality rates among Hispanic and non-Hispanic white women shows that rates have continued to decline in both ethnic groups, but the rates of decline have been disparate, with a 39% reduction in mortality among white women compared with only a 29% reduction in mortality among Hispanic women.^4^ Furthermore, Hispanic women presented with more locally advanced disease than non-Hispanic White women.^11^ However, the most pronounced mortality gap exists between white women and black women, who remain 30–40 % more likely to die after a diagnosis of breast cancer.^1–3,12^ In addition, black women have a higher incidence of breast cancer diagnosed before the age of 45 years and a higher risk for diagnosis of more aggressive disease (e.g., triple-negative breast cancer [TNBC]) than all ethnic groups and across all age categories.^1^

This review summarizes research that has contributed to our understanding of disparities in breast surgical oncology across all racial/ethnic groups. However, given that the most egregious disparities exist among black women, our review involves considerable focus on this racial group. We discuss racial disparities across the breast cancer continuum including access to care, diagnosis, surgical treatment, and survivorship and conclude with strategies to redress them.

## Disparities in Access to Care

Implementing access-enhancing strategies and reducing barriers can improve health care outcomes significantly among racial and ethnic minorities.^13^ Fayanju et al.^14^ previously described the under-utilization of three-dimensional (3D) mammography (i.e., Tomosynthesis) among non-white women and have strongly recommended that all women undergo breast cancer risk assessment by a primary care provider (PCP) or OBGYN before the age of 25 years to ensure that all women begin screening at an appropriate age based on their overall risk for the development of breast cancer. Particularly, health insurance coverage for low-income adults has been associated with improvement in access to care, consistent use of preventive health services, and improved self-reported health status.^13,15^

Among patients with comorbid and chronic conditions, improved health insurance coverage is linked to medication compliance and improved patient-physician communication.^15^ Both Medicaid expansion and the Centers for Disease Control and Prevention (CDC) Breast and Cervical Cancer Control Program^16,17^ have been associated with improved access and guideline-concordant time to treatment. Conversely, uninsured or under-insured status is associated with worse health outcomes.

In a 10 years retrospective review focusing on overall survival and relative risk of death among female patients with early-stage breast cancer, Jemal et al.^18^ noted that black and Hispanic women were three times more likely to be uninsured or to have Medicaid (22.7%) than white counterparts (8.4%). Lack of insurance resulted in a 33% excess risk of death, specifically among nonelderly black patients with early-stage breast cancer.^18^

Lack of access also is confounded by structural racism, which is defined by the deeply rooted complex barriers whereby society reinforces racial discrimination through resource distribution and inequitable systems for health care delivery.^6^ To combat the effects of structural racism and create access-enhancing solutions, it is necessary to understand the historical origins and modern-day implications of inequitable distribution of high-quality health care, the financial toxicity related to treatment, medical mistrust and the lower levels of health literacy among minoritized individuals.^14^

Due to the deeply ingrained structural racism affecting all aspects of health care delivery, mitigation requires population-level policy reform.^6,13–15,18–20^ At an individual level, training physicians and health care providers about dismantling structural racism, counteracting interpersonal discrimination, and focusing on cultural humility and perspective are mechanisms that can help us collectively achieve health equity.^6^

### Recommendations

Equitable health care is limited by the effects of structural racism, which reduces access to care, promotes inequitable distribution of resources, increases medical mistrust, and limits health literacy. To mitigate the effects of structural racism, reducing barriers to access such as lack of insurance, implementing patient education materials designed for diverse levels of health literacy, and focusing on training culturally sensitive physicians dedicated to policy reform are critical.

## Differences in Histologic Subtype

Histologic subtypes and associated survival outcomes contribute but do not wholly explain the increased breast cancer-specific mortality seen among black women.^21^ Triple-negative breast cancer (TNBC) is characterized by non-expression of the estrogen receptor (ER), progesterone receptor (PR), and human epidermal growth factor receptor 2 (HER2) and accounts for 25% of breast cancer-related deaths despite representing only about 10% of breast cancers in the United States.^1^ However, it is more common among women of African ancestry, among whom 15 to 30% of breast cancers are TNBC.

Some germline genetic patterns among African American women have been linked to West African ancestry and associated with the increased rates of TNBC seen in this racial group.^22^ A population-based study by Lund et al.^23^ focusing on associations between race and TNBC showed TNBC to be more prevalent among African American females younger than 50 years (odds ratio [OR], 1.9; 95 % confidence interval [CI], 1.2–2.9) after adjustment for age, grade, stage, and the multidimensional poverty index (MPI). To assess poverty comprehensively, the MPI measures the percentage of households in a country with poverty defined along three axes (financial poverty, education, and basic infrastructure services). Importantly, mortality also was higher for black women with TNBC (hazard ratio [HR], 2.0; 95% CI 1.0–3.7), particularly given that no targeted therapies against TNBC are available currently.^24,25^

Conversely, hormone receptor-positive/HER2-negative breast cancer carries a more favorable prognosis, with targeted treatments designed to inhibit ER-mediated pathways, although the advent of immunotherapy has improved rates of pathologic complete response (pCR) after neoadjuvant systemic therapy as well as event-free and overall survival.^26^ However, in literature focused on understanding racial disparity in ER-positive breast cancer, a small population-based study in Illinois noted a four-fold increase in risk of death for black patients with a diagnosis of ER/PR-positive breast cancer after adjustment for stage, grade, and time to treatment (HR, 4.39; 95% CI, 1.76–10.9), suggesting a multifactorial etiology.^1,8,27^

Deviations from standard chemotherapy treatment doses also have been specifically and widely observed. Griggs et al.^25^ compared initial chemotherapy dosages among 500 women at 10 treatment sites across two geographic regions with standard initial dosages and found that black women received lower chemotherapy doses, which were associated with lower treatment intensity than whites experience. Furthermore, evidence exists to show that much of the overall racial disparity observed in mortality between black women and white women actually is driven by differential mortality from hormone receptor-positive breast cancer, specifically luminal B tumors, for which, in contrast to TNBC, several effective therapies already exist.^28^

Luminal cancers comprise almost 70% of all breast cancers. Both luminal A and B tumors are typically ER+, but luminal B breast cancer is more aggressive than the luminal A type due to more common low/negative PR expression, high proliferation, higher grade, and predicted poorer response to endocrine therapy. Luminal B breast cancer is more prevalent among black women with breast cancer than among white women, and suboptimal recognition and potential undertreatment of this intrinsic subtype is potentially a contributor to racial disparities in survival after breast cancer diagnosis.^29^

### Recommendations

Given the higher rate of TNBC among black women, additional clinical trials with diverse enrollment and a focus on targeted agents are critical as are efforts to provide equitable and standardized treatment for all subtypes of breast cancer.

## Disparities in Surgical Treatment

Surgery is the first treatment for a majority of patients with breast cancer, and treatment initiation longer than 60 days after diagnosis is associated with worse survival.^30^ Although discussion of systemic and radiation therapy disparities is beyond the scope of this review, it is important to note that differential prescribing patterns and treatment delays for these treatments further compound disparities in mortality.^13^

Disparities in time to surgery, surgical outcomes, and postoperative complication rates have been described in the literature. Chavez-MacGregor et al.^31^ noted factors associated with these delays in a retrospective review of patients with a diagnosis of early-stage breast cancer. Factors associated with delays in time to treatment included low SES, non-private insurance, Hispanic ethnicity, non-Hispanic black race, and reception of breast reconstruction.^31^ Longer time to treatment resulted in worse overall survival among women with TNBC (HR, 1.53; 95% CI, 1.17–2.00).^31^ Similar findings were noted by Fedewa et al.^32^ studying delays in administration of chemotherapy associated with insurance status, stage, and comorbid conditions. Both black and Hispanic patients were at higher risk than white patients for a treatment delay longer than 60 days (relative risk [RR], 1.36 [95% CI 1.30–1.41] and RR 1.31 [95% CI, 1.23–1.39], respectively) as well as a 90 days treatment delay (RR, 1.56 [95% CI, 1.44–1.69] and RR 1.41 [95% CI, 1.26–1.59], respectively).^33^

In a retrospective cohort analysis focused on longer time to surgery for Asian American/Pacific Islander women than for white women with stages 0 to III breast cancer, Patel et al.^9^ noted that the median time from initial diagnosis until the first day of surgery was 31 days for white women, 34 days for East Asian patients, 34 days for Pacific Islander patients, 36 days for Asian Indian or Pakistani patients, and 38 days for the Southeast Asian patients. In addition, Asian American/Pacific Island patients were more likely than white patients to present with stage II or III disease (35.63% vs 34.43%; *p *< .001).^1^ When comparing time to initiation of treatment across all racial groups at an academic cancer center, Patel et al.^9^ noted that Hispanic women had the longest median length of time between diagnosis and treatment initiation (71 days) compared with Asian/Pacific Islanders (51 days), blacks (47 days), and whites (45 days) (all *p *< .001). In addition, these authors noted that Hispanic patients also were more likely to report practical (i.e., logistical) contributors to distress, suggesting the important role that proactive ascertainments of patients’ social determinants of health (SDOH) and screening for unmet social needs can play in potentially preempting disparities.^34^

A study examining surgical interventions and outcomes among 71,156 women hospitalized with a primary diagnosis of breast cancer noted that black women experienced more post-surgical complications (OR, 1.21; 95% CI, 1.04–1.42), and more in-hospital mortality (OR, 1.26; 95% CI, 1.07–1.50) than white women even after adjustment for comorbidities and treatment type.^35^ Asian, black, and Hispanic women are less likely than white women to undergo post-mastectomy reconstruction, and among recipients of reconstruction, postoperative complication rates appear to be higher among black women than among white women.

A review of the National Inpatient Sample by Sarver et al.^36^ studying perioperative post-mastectomy complication rates showed black women undergoing autologous reconstruction more frequently (40.7 vs 28.3%), experiencing more perioperative comorbidities (e.g., diabetes: 12.9 vs 5.8%), and having higher Charlson-Deyo combined comorbidity scores ( ≥ 4%: 5.5% vs 2.7%) than white women. The authors posited that suboptimal primary care access resulting in poor preoperative health optimization of comorbid conditions may compound postoperative health complications, resulting in longer hospital stays.^37^

**Fig. 1:**
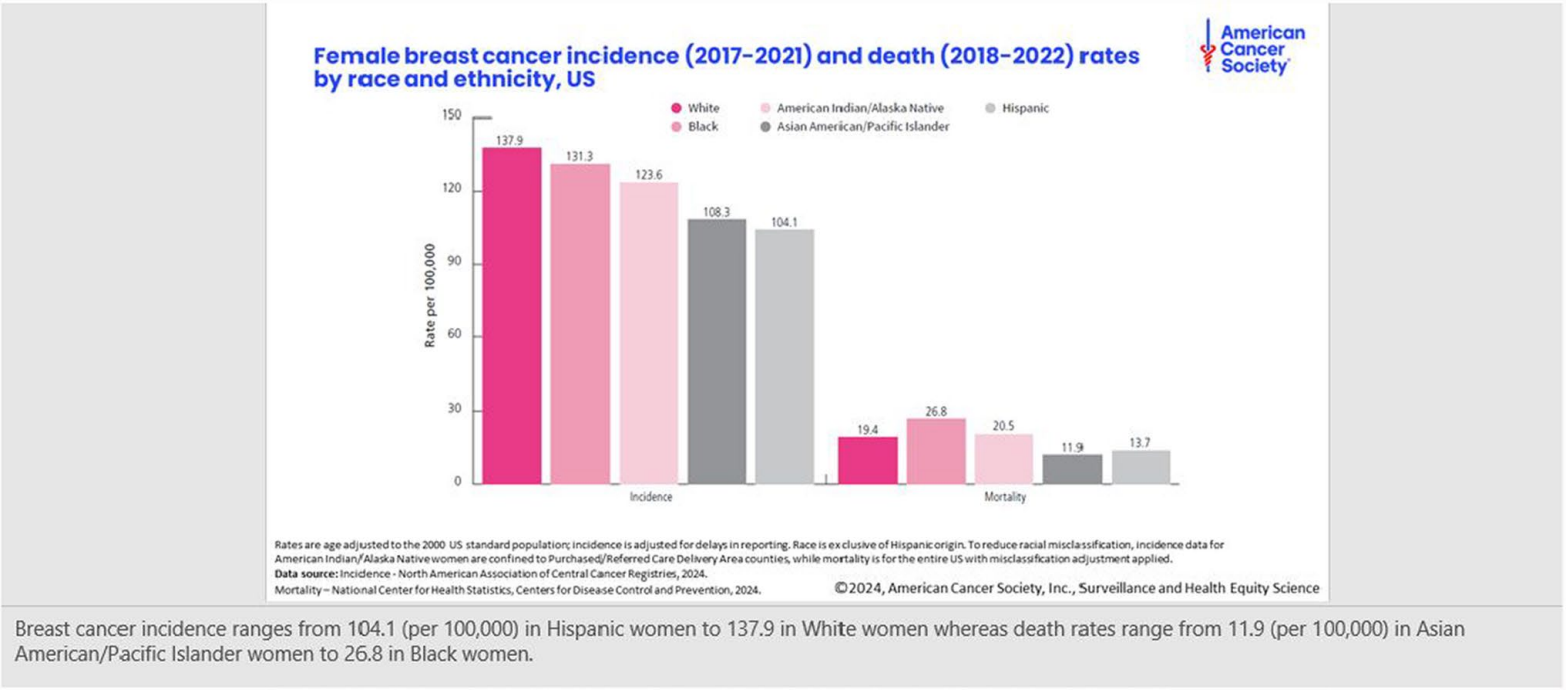
Female breast cancer incidence and death rates by race and ethnicity in the United States.^1^

In a retrospective review of 1045 patients undergoing breast reconstruction in which comorbid conditions were compared with postoperative complications, Hispanic and black patients had higher rates of arterial and venous insufficiency and post-mastectomy necrosis than white women (34.1 % vs 27.4 %; *p* = 0.021).^37^ In contrast, Butler et al.^37,38^ found in their retrospective review that black patients had medical and surgical outcomes after autologous free-flap breast reconstruction compared with those of white patients despite higher levels of comorbidities and risk factors.^38,39^ However, the literature has shown that even in equal-access health care settings and in clinical trials with standardized treatment and care, significant racial disparities still exist despite control for comorbid conditions (Fig. 1).

Importantly, Morrow et al.^34^ noted that black patients with breast cancer had higher rates of postoperative dissatisfaction, even after control for SES, education level, or reconstruction (OR, 2.87; 95 % CI, 1.27–6.51), experiences further compound medical mistrust.^34^ Shared decision-making models focusing on a positive physician-patient relationship and patient education can bolster overall patient satisfaction. A systematic review focusing on effective shared decision-making models by Bomhof-Roordink et al.^40^ cites two key factors in achieving patient satisfaction with shared decision-making: reaching mutual agreement about the desired outcome and framing discussions on the process rather than the final treatment result.

### Recommendations

Preoperative medical optimization in collaboration with primary care providers can be an effective approach to identifying and optimizing patients who may be at risk for treatment-related complications associated with comorbid conditions. Key interventions including preoperative patient education, improvement in primary care access, and shared decision-making are recommended to improve satisfaction.

## Disparities in Survivorship and Palliative Care Access

Survivorship focuses on long-term health outcomes and surveillance after completion of surgery, chemotherapy, and/or radiation treatment.^41^ Surveillance mammography plays a vital role in supporting survivorship. In a 20 years systematic review of racial demographics associated with surveillance mammography after breast cancer treatment, Advani et al.^42^ noted that non-white women survivors of breast cancer were less likely to receive surveillance mammography than white women.^41^

Another important component of survivorship is health maintenance, associated with improved co-management of non-breast cancer-related comorbid conditions with PCPs.^42,43^ Specifically, obesity is associated with increased risk of breast cancer recurrence.^43^ Advani et al.^42^ conducted a study of 17,158 cancer survivors and found that black and Hispanic survivors were more likely to be overweight or obese than non-Hispanic white survivors.^42^ These results have been replicated in numerous additional studies.^44–46^ Given the success of group-based health maintenance programs, survivorship should ideally also include equitable and accessible population-based health interventions at the community level.^43^

Palliative care plays a critical role in the management of peri- and postoperative pain, management of symptoms, end-of-life decision-making and has been shown to improve cancer patients’ quality of life.^47–50^ Disparities also exist in access to palliative care resources among Hispanic and black breast cancer patients with metastatic disease. Freeman et al.^51^ conducted a 16-year retrospective study of 148,931 patients with metastatic breast cancer focusing on access and survival differences among various racial groups. Among palliative care users, black patients had lower palliative care use and a greater mortality risk then all other ethnicities (HR, 1.14; 95 % CI, 1.07–1.21), whereas a lower mortality risk was found among Asian (HR, 0.83; 95 % CI, 0.71–0.97) and Hispanic (HR, 0.77; 95 % CI, 0.69–0.87) patients. Lee et al.^52^ conducted a retrospective cohort study analyzing 204,175 inpatient oncologic palliative care consultations and noted that black patients were significantly less likely to receive a palliative care consultation than white patients (OR, 0.69; 95 % CI, 0.62–0.76).

Even among those patients who received palliative care referrals, multiple studies have further demonstrated additional surgical palliative care disparities, noting physician bias that resulted in delays to referral and led to more disease progression before initiation of palliative care.^47–51^ Despite oft-cited concerns among patients and providers that invocation of palliative care signifies resignation toward death and deviation from treatment that is primarily curative in its intent, the actual result of these bias-related care patterns is that black patients are less likely to be discharged from the hospital alive.^53,54^

### Recommendations

Access to high-quality primary care is necessary to mitigate comorbidities, prevent recurrences, and assist patients with surveillance imaging after treatment. Educating patients and providers about palliative care as a resource-intensive approach for goals of care alignment can reduce stigma and improve access via early dissemination of resources. Preempting patient bias, prioritizing clear communication, and providing early palliative care consultation also may assist with end-of-life comfort, ensure resource provision, and even extend survival.

## Summary and Future Directions

Factors associated with health disparities in breast cancer remain complex and multifactorial. In addition, whereas this review extensively discussed only black patients with breast cancer, disparities continue to exist among multiple racial groups including Asian, Hispanic, and indigenous patients as well as patients of all racial groups with lower SES.^55^ Solutions to close gaps must be enacted at the levels of clinicians, institutions, and policymakers in collaboration with patients and should include the following:

1. Early breast cancer risk assessment (before the age of 25 years) should be conducted by PCPs and OBGYNs to identify women at elevated risk for breast cancer who should benefit from early and enhanced screening.
2. Tomosynthesis (i.e., 3D mammography) should be implemented universally for breast cancer screening, with prioritization of timely diagnostic resolution for patients with abnormal imaging who are at elevated risk for loss to follow-up evaluation (e.g., mobile mammography participants, black women).
3. Diverse clinical trial participation is necessary to provide equitable and generalizable screening and treatment recommendations for all racial groups. In particular, there needs to be an increased focus on identifying and elucidating the pathophysiology of therapeutic targets for aggressive breast cancer subtypes such basaloid/triple-negative and luminal B that contribute disproportionately to breast cancer-related death, particularly among Hispanic and black women.
4. Bi-directional communication and collaboration between oncologic and primary care providers can help optimize pre-existing medical conditions, concomitantly reducing the likelihood of treatment-related complications and decreasing the likelihood of delayed or omitted care because of these conditions.
5. Clinicians should be instructed on how to optimize therapeutic relationships with diverse patients via shared decision-making, and educational materials should be provided that meet patients across a wide spectrum of health literacy.
6. Improving access to insurance via policy-level reforms (e.g., Medicaid expansion, the CDC Breast and Cervical Cancer Control Program) and institutional provision of financial counselors can help improve equitable initiation of guideline-concordant care and reduce financial toxicity.
7. Institution-level bias-reduction models must target the multifactorial etiology of systemic racism to create a framework of equity-centered care.
